# Understanding Youth’s Lived Experience of Anxiety through Metaphors: A Qualitative, Arts-Based Study

**DOI:** 10.3390/ijerph18084315

**Published:** 2021-04-19

**Authors:** Roberta Lynn Woodgate, Pauline Tennent, Nicole Legras

**Affiliations:** Rady Faculty of Health Sciences, College of Nursing, University of Manitoba, 89 Curry Place, Winnipeg, Manitoba, MB R3T 2N2, Canada; Pauline.Tennent@umanitoba.ca (P.T.); Nicole.Legras@umanitoba.ca (N.L.)

**Keywords:** youth, mental illness, anxiety, depression, metaphors, qualitative research, arts-based research methods, open-ended interviews, photovoice, ecomaps

## Abstract

Living with anxiety can be a complex, biopsychosocial experience that is unique to each person and embedded in their contexts and lived worlds. Scales and questionnaires are necessary to quantify anxiety, yet these approaches are not always able to reflect the lived experience of psychological distress experienced by youth. Guided by hermeneutic phenomenology, our research aimed to amplify the voices of youth living with anxiety. Fifty-eight youth living with anxiety took part in in-depth, open-ended interviews and participatory arts-based methods (photovoice and ecomaps). Analysis was informed by van Manen’s method of data analysis with attention to lived space, lived body, lived time, and lived relationships, as well as the meanings of living with anxiety. Youth relied on the following metaphors to describe their experiences: *A shrinking world; The heavy, heavy backpack; Play, pause, rewind, forward; and A fine balance.* Overall, youth described their anxiety as a monster, contributing to feelings of fear, loss, and pain, but also hope. The findings from this study can contribute to the reduction of barriers in knowledge translation by encouraging the use of narrative and visual metaphors as a communicative tool to convey youth’s lived experience of anxiety to researchers, clinicians, and the public.

## 1. Introduction

The burden of mental illness for young people across the world is growing in magnitude [1], now more than ever [2], and especially as they find themselves disproportionately affected by the COVID-19 pandemic [3]. A 2015 meta-analysis revealed a significant global prevalence of anxiety (6.5%) and depressive disorders (2.6%) among children and adolescents [4]. In Canada, the diagnostic rate of anxiety disorders in youth (ages 12–24 years old) doubled in 2018 compared to 2011 (12.9 vs. 6.0%, respectively) [5]. Anxiety is manifested in many forms, with comorbidity between anxiety disorders [6,7] and/or depression very common [8]. Upwards of 50% of adult mental health disorders have their onset during adolescence [1], making timely and effective intervention key to lessening the burden of illness over the long term [9,10]. The need for context-specific understandings of the mental health experiences of adolescents has been identified as a one of the major gaps in global research [11]. Measures such as scales and questionnaires are necessary to quantify anxiety, yet these approaches risk not adequately reflecting the lived experience of psychological distress across individuals and settings [12]. Living with a mental illness, including anxiety [13] and depression [14], is a complex, biopsychosocial experience that is unique to each person and embedded in their contexts and lived worlds. Given the breadth and complex intricacies of mental illness experiences, a qualitative approach presents several advantages [15]. Our research aimed to amplify the voices of youth living with anxiety and highlight salient metaphors (narrative and visual) of the lived experience of anxiety that make knowledge more available and applicable to others.

According to Riceour’s *The Rule of Metaphor*, metaphorical language was first described by Aristotle as “giving the thing a name that belongs to something else” [16]. This decorative function is aligned with the traditional theory where metaphors are used to embellish language while saying exactly the same thing [17]. However, Aristotle further explained that metaphors can also be used when there is no existing word for an object to create new ways of understanding it [18]. In this function, metaphors are used to “express and (…) deal with perceived phenomena” [19]. All metaphors in the most basic form comprise three aspects: “the idea of a deviation from ordinary usage, the idea of borrowing from an original domain, and the idea of substitution for an absent but available ordinary word” [18]. Essentially, metaphors re-describe a concept in terms of another concept [20,21,22,23] by highlighting the salient features of a concrete, readily understood, familiar domain and exporting them into a more complex, abstract, unfamiliar domain to help people to organize the information in their minds [24,25]. The contemporary theory pioneered by Lakoff and Johnson [26] elaborates on the cognitive processes used by individuals to interpret language and holds that metaphors define and reflect deeply entrenched cultural understandings [27,28,29]. Members of a “given group of people” [30] share a cultural context, a linguistic code, and backgrounds (e.g., historical, social, professional) [31] have the same *collective cognitive models* [17], meaning they are more likely to have a similar understanding of a metaphor. In addition, metaphor interpretation is also shaped by personal conception and experiences, considered part of our individual *partial cognitive models* [19]. Together, these cognitive models explain how metaphors function as “contextual renegotiations” [32] of language to create new ways of understanding [17].

Discussions of metaphor use in illness communication can be traced back to Sontag’s work on cancer, tuberculosis, and AIDS, where she argued against the use of metaphors due to concerns of negative public and patient perception of illness stemming from their use [33,34,35]. Despite differing recommendations regarding the “best” metaphors to use in medicine, the truth is that no metaphor is “inherently good or bad…each is contextual” [36,37]. The literature supports the use of metaphors as a communication tool for clinicians to enhance patients’ comprehensibility [38], and inversely for patients to meaningfully express themselves [36]. Metaphors can access and symbolize emotions that were previously elusive or too difficult for the patient to express directly [39,40,41]. The facilitation of self-expression using metaphors is important for meaning making as patients seek to reconcile the existential with the experiential during their illness journey [19,39,42]. In this way, metaphorical language stabilizes chaos and instils a sense of control by capturing what is impossible to describe literally [42,43]. Across illness experiences, metaphors have proven to be essential to the way patients make sense and communicate about their illness, including its physical, emotional, and psychological implications [42,44,45,46,47,48,49].

With regard to mental illness specifically, the literature has focused on patients’ use of metaphors in narratives of distress, well-being, and recovery [45,50,51], as well as metaphor use in public messaging about mental health [52,53]. Metaphors provide a compact, vivid portrayal of an experience [39], acting as a “gateway into the perceptual world of others” [19], especially for experiences that are outside the “normal” frame of reference for many, such as addiction [49,51]. Metaphors that occur in multi-modal formats (e.g., static or moving pictures, music, sounds) can highlight the most salient features of the concept simultaneously to best promote understanding [54,55]. Visual metaphors in particular have shown to be an effective strategy to communicate about the lived experience of mental illness [52]. Photovoice as a form of visual metaphors has helped to convey hard-to-conceptualize experiences of suffering, stigma, and recovery in individuals with a mental illness [56,57]. It is clear that metaphors are a helpful communication tool both for patients as they share their illness narrative with others [58] and for their audience as they seek to understand an experience “for the first time or in a novel way” [59]. Realizing the potential of metaphorical language in healthcare communication can contribute to bridging the gap between the patient’s lived experience, the clinician’s medical framework, and the public perception of an illness [60,61,62]. Our study aims to provide a platform to amplify youth’s lived experience of anxiety, highlighting their use of metaphors as a communicative tool with the ability to translate knowledge across barriers between researchers, clinicians, and members of the public.

## 2. Materials and Methods

### 2.1. Research Design

Hermeneutic phenomenology—a research philosophy [63] and qualitative methodology [64]—guided the “Youth’s Voices: Their Lives and Experiences of Living with an Anxiety Disorder” research study. Hermeneutic phenomenology attempts to make sense of real-life experiences as they are lived by individuals in their social worlds, recognizing that these understandings are always interpreted and contingent [65]. The goal of hermeneutic phenomenology is to describe the meaning of an experience, including *what* was experienced, and *how* it was experienced [66]. As guiding concepts, hermeneutic phenomenology is concerned with lived meaning—the way that a person experiences and understands their world as real and meaningful; and lifeworld—how that life is experienced in the context of being intertwined with the world [64,67]. Van Manen’s lifeworld includes four existentials—lived time (i.e., the ways individuals temporally experience their lifeworld), lived space (i.e., the ways people experience the spatial dimensions of their daily experiences, including feelings of being in place), lived body (i.e., the bodily experience of everyday life including how we feel, reveal, conceal, and share), and lived human relations (i.e., the relations we make and maintain) [68].

### 2.2. Participants

In total, 58 young people from Winnipeg, Manitoba, Canada, with a primary diagnosis of one or more anxiety disorders participated in the Youth Voices project. This included separation anxiety, specific phobias, panic disorder, agoraphobia, social anxiety disorder, and generalized anxiety disorder. Participants experienced anxiety and other co-morbid conditions (e.g., depression) ranging from moderate to severe. As an exclusion criterion, youth who had a confirmed diagnosis of either obsessive-compulsive disorder (OCD) or post-traumatic stress disorder (PTSD) were not included in the project as these disorders present differently with different symptoms. Overwhelmingly, participants stated that they felt they had lived with traits of anxiety for many years, prior to a specific diagnosis. We also interviewed parents/caregivers of youth participants. While not presented as part of the findings of this paper, the purpose was to explore their perspectives of parenting a youth with anxiety and its impact on the family.

The selection of participants followed the maximum variation technique of purposive sampling [69] with the aim to include a diverse range of information-rich experiences [70]. While 58 is a large number of participants for a phenomenological study, it was important to gain a comprehensive understanding [71,72,73] given that this was one of the first research projects in Canada that aimed to detail the lived experience of young people with anxiety. The research team had the resources necessary to manage this large sample size so as to permit deep and robust engagement with the data.

Youth were recruited from both clinical (e.g., via invitation letters sent to patients on the waitlist for hospital-based programs that specialize in the treatment of anxiety disorders) and community (e.g., social media, youth centres, schools, and teen clinics) settings. The severity of youth’s anxiety was confirmed by parents and/or youth participants. There was a great deal of interest in this research study, with many youth participants willing to “push through” any social anxiety they may have been experiencing, in order to participate in this project. Youth participants ranged in age from 10 to 22 years of age at the time of the study (M = 14.5 years). The overwhelming majority of participants in the project identified as female (N = 44) and white (N = 40).

Ethical protocols included a review from the university’s research ethics board, ethics review from the collaborating healthcare settings, written prior and informed consent and assent, and an ongoing process of verbal consent. Participants received an honorarium in appreciation of their time and expertise.

### 2.3. Data Collection

All youth participants took place in qualitative, open-ended, in-person interviews. In-depth, open-ended interviews presented an opportunity to gather rich and detailed descriptions of the meaning and experience of living with an anxiety disorder [70,74]. The majority of youth participated in repeated interviews, to the extent that they were able. Such an approach—an essential feature of a hermeneutic phenomenological design—allowed for follow-up questions to expand on the content identified in the initial interview, while also exploring new topics [75].

An interview guide with open-ended questions was developed in collaboration with the research team and then refined further through the data collection process. The first interview was also supplemented by asking youth participants to create an ecomap—a graphic portrayal of social relationships or networks of individuals or families, as well as places and activities [76]. Prior to asking youth questions about their experiences of living with an anxiety disorder, they were asked to draw nodes that represent people, activities, and places that are and have been a part of their lives. Youth were asked to indicate the degree of connection that they have with each person, activity, or place represented on the map (using lines, colours, or another means). Different types of lines and/or colours represented different types of connections. The ecomaps were used throughout the first interview to promote discussion. Additional interview strategies included the use of silence, the elicitation of examples, and probes to expand on something the participant had said [77]. When probing youth, great care was taken to ensure that the questions remained close to youth’s experiences. At the end of the interview sessions, participants were given the opportunity to add questions to the interview guide. In one such case, a 13-year-old participant requested that we add the question “what does anxiety look like in your head?” to our interview guide. This metaphorical nature of this question generated a number of responses included in this paper; however, other metaphors were offered by participants unprompted.

The interview process was complemented by the arts-based methodology of photovoice. Photovoice is a participatory method that involves individuals taking photographs to document the issues most significant to them [78,79], as well as engaging in critical reflection and dialogue about those images [80]. As an arts-based method, photovoice embodies a phenomenological research design, offering a direct expression of lived experiences, through alternative (i.e., visual) means [81,82]. For this project, photovoice did not take place in the group setting as is common. Rather, the researcher introduced photovoice to individual participants following their first research interview, who then worked on their individual photovoice project over the course of approximately four weeks, with the researcher checking in with them. A second individual open-ended interview was scheduled with youth participants, offering an opportunity for the researcher to follow up on the first interview and generate thick description of their lived experience. Also, in the second interview, the SHOWeD technique—as commonly used in photovoice [78]—was used by the researcher as a tool to generate discussion with each research participant as to the images they took and selected for the project. The technique consists of a series of questions posed to participants regarding their selected images including: What do you **S**ee here?; What is really **H**appening here?; How does this relate to **O**ur lives?; **W**hy does this problem/concern/strength **E**xist?; and What can we **D**o about it? [80,83].

All interviews were conducted in person at a private location that was most convenient and comfortable to participants. Interviews ranged in length from 30 min to 3 h. After each interview, field notes were recording detailing the context of the interview. All interviews were digitally recorded and transcribed verbatim to preserve their authenticity. 

### 2.4. Data Analysis

Hermeneutic phenomenology recognizes that the development of meaning and knowledge occurs within the interactions between the researcher, the research participant, the research question, as well as the broader environment in which the research takes place [84]. Data analysis occurred alongside data collection, in an iterative approach that allowed research participants to have ongoing input into the development of themes. As informed by van Manen’s method of data analysis [64], all data emerging from the project (including interviews, ecomaps, field notes, and photographs) were included in the analysis. Data were read (and re-read) with attention to significant statements, sentences, or sentence clusters that stood out as thematic of the lived experience of youth with anxiety disorders. Through multiple analytic discussions with the first author, research team members, and research assistants, units of meaning were delineated and formed into thematic statements, and themes were extracted. Graphic data (i.e., ecomaps and photographs) helped to inform the themes emerging from the youth interview data, serving as graphical representations of the text-based findings, which contributed to a greater understanding of the experiences of youth [85].

For knowledge translation (KT) activities, we took direction from youth participants who in the context of interviews shared that it was important to them that the research from this project be used in a way that raised awareness of what it was like to be a young person living with anxiety. Through discussions with and the guidance of a Youth Advisory Committee, film was identified as an exciting opportunity that could do just that. We worked with a local film company to develop two film series [86,87] that amplified the voices of young people. One of the series featured choreography [87], while the other utilized line animation [86]. Moreover, an online zine with some of the photovoice submissions was developed. Every word in each of these three KT products came directly from the interview transcripts with young people, with their photovoice images used as further symbolic inspiration (see Supplementary Materials).

## 3. Results

Youth often struggled to find the words to describe the ways in which anxiety plays out in their everyday lives. When asked to share their experiences of living with anxiety, a 14-year-old youth first attempted to describe some of the emotions involved, but felt that the words alone were insufficient and resorted to metaphor:

When I get like really anxious…like I think I’m kind of using anger as a way to explain it even though it’s not exactly it…. Like I said I get angry a lot which I guess I do sometimes, but it’s not exactly anger it’s just something I can’t really describe…. It’s like a feeling, it’s kind of like a mix between anger and like apathetic, like apathy to myself and like stuff like that. It’s just kind of like a mix of a bunch of things that like making my mood kind of like [long sigh]. It’s [anxiety] like when you put a cat inside a bag and they start like clawing at it, I don’t know.

To help generate understanding around the experiences of living with an anxiety disorder, youth relied on metaphors to describe their experiences and how they navigate the world, with attention to Van Manen’s theoretical concepts of lived space, lived body, lived time, and lived relationships. Youth also used photovoice as a metaphor itself, with the photovoice process allowing them to make representations of their anxiety and its manifestations that could be shared with others in order to generate understanding. We discuss the metaphors based on van Manen’s four existentials. After presenting this information, we further present metaphors that reflect on the meanings of living with anxiety. These overall meanings—of fear, of loss, of pain, but also of hope—were shaped by van Manen’s four existentials.

### 3.1. The Metaphor of Lived Space: A Shrinking World

Lived space encompasses how individuals experience space, as well as how space affects an individual’s subjectivity [88]. Overall, youth living with anxiety disorders spoke of a shrinking world to describe the ways in which their lives were limited as a result of their anxiety. A 16-year-old participant shared:

It’s [living with anxiety] like living in a box, and the box keeps on going smaller and smaller every single day…To me in my head when I first think of anxiety it’s like you’re in an empty world and there’s nothing, no one’s around you, you’re trapped in this world that you’re trying to get, you’re screaming for help and no one can hear you…. And it feels like that you’re getting closer to the edge of like of things, you feel like you’re getting closer to the edge of the world and everything and no matter where you walk or step or whatever you do it’s like the world is getting like getting more surrounding you.

**Figure 1:**
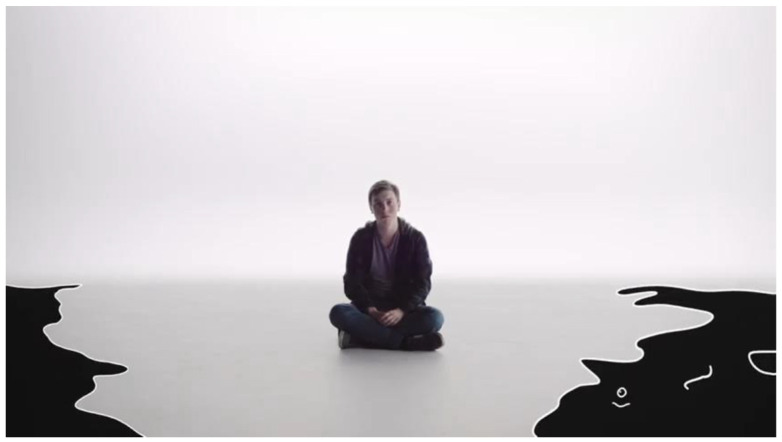
Still image of black sludge from *The Monster*, inspired by the above quote from a participant.

Throughout the narratives, different participants described their experiences of living with anxiety as being similar to “drowning”, and as feeling as if they were “stuck…can’t go anywhere or do anything.” The language utilized by youth depicts an image of a world that is closing in on them. Youth relied of spatial metaphors to embody feelings of being trapped and being stuck in place. A 17-year-old participant described living with anxiety with the following analogy that featured prominently in our film *The Monster* [86] (Figure 1):

Anxiety is like lot of black sludge…Like I see myself sinking a lot of the time. And that’s a common image that I’ve looked at throughout my poetry… I wrote about it in my journal and it’s just a bunch of fears and stuff that I was upset about and really what anxiety and depression look like to me is just black sludge.

**Figure 2:**
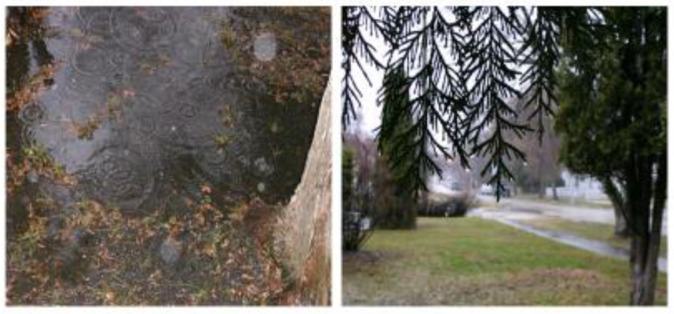
“Gloomy and grey”.

In their descriptions of living with anxiety, youth spoke of specific spaces (e.g., schools, bedroom), as well as sights and sounds of lived spaces (e.g., busy streets, the noise of traffic, the noise of construction) as being representative of their anxiety. For some youth, these spaces further describing the feeling that their worlds were closing in on them. In general, youth described their social worlds as being a dark place, such as the following quote to describe a photograph (Figure 2) taken by a 16-year-old:

With anxiety you kind of feel like it’s always like rainy and gloomy and grey and just like, like with anxiety sometimes because it does, it can disable people to do things. Everything is just grey, and you can’t see the sun and everything kind of sucks.

Because their lived space was perceived as dark and shrinking, many young people described their challenges as they tried to navigate that lived space. Some youth spoke of the challenges they experienced having to leave those spaces they considered safe (e.g., their homes) to go to spaces considered less safe, including those known (e.g., school) and unknown (e.g., public transportation, get together with friends).

**Figure 3:**
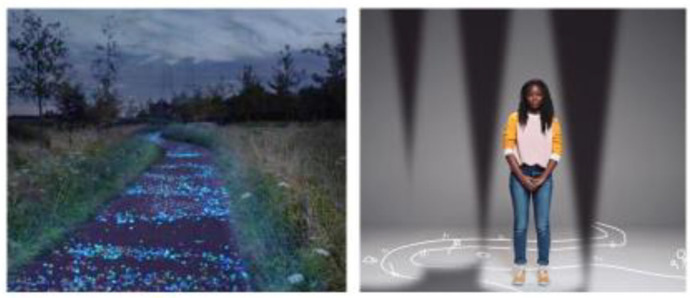
Participant’s photovoice submission of a screenshot of a “lit path”, alongside still from the film *Fighting to Stay on the Path* featuring the artists’ interpretation.

Youth spoke about the need to navigate through their lived spaces in spite of their anxiety, highlighting their resiliency. In the context of photovoice, a 16-year-old shared an image of a screenshot (Figure 3) taken from the internet and explained its significance to her. The participant’s quote became a key element in our film *Fighting to Stay on the Path* [87] as the still image demonstrates:

This is a screenshot of a railway track with, with pebbles or flowers but some of it glows in the dark and I just thought the idea that like this path that you’re on it can be surrounded by darkness and fear but if like the path itself is like lit and I don’t know I just saw it as like no matter what is horrible or scary or that makes you fear yourself or others around you like as long as you stay on this path and you keep going it’s like a beautiful journey and like as long as you keep going on the path it’s going to keep you safe…. Cause that’s what I see, like the path is the only thing that looks safe in this like field or that looks secure and it looks lit.

### 3.2. The Metaphor of the Lived Body: The Heavy, Heavy Backpack

Lived body refers to bodily experience in everyday life, including how we feel, reveal, conceal, and share those experiences [88]. Overall, youth described their anxiety as a heaviness in both their mind and their body. They spoke about the weight of their anxiety, which led to feelings of exhaustion and being depleted of energy. One youth described living with anxiety with repeated reference to a backpack:

…you’re crawling instead of just having that stroll to achieve whatever, you’re almost crawling and almost scraping through the floor, like trying to get there…. Cause anxiety is like riding on your back almost. And you’re like crawling and it’s so heavy…. And like you know I don’t need this luggage, I can stand up straight and get this done…. But it’s just, I guess anxiety is that burden, the heavy heavy backpack on your, its full of things you don’t even need but they are there.

**Figure 4:**
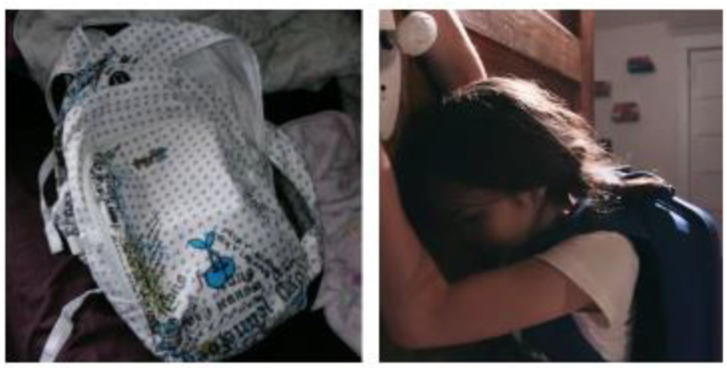
Photovoice submission submitted by youth participant alongside still image from *Can’t You See I’m Struggling?*

This reference to a backpack also featured in other participants’ photovoice submissions (Figure 4), with similar descriptions as noted above. As such, we then incorporated the symbolic nature of the heavy backpack into our film series, with each performer carrying a backpack throughout the vignettes [87].

Youth also relied on metaphors to describe living with symptoms of their anxiety. Many participants described the magnitude of living with anxiety as an assault on the body. A 17-year-old who described experiencing panic as one of her symptoms of anxiety, went on to share metaphors that described both the physical and mental aspects of living with an anxiety disorder but also ended her comment struggling to find the words:

Well when my breathing’s acting up [with anxiety] it feels like, you know how like you’re underwater and your lungs start to get tight…. That’s what it kind of feels like for me…But and then with the guilt like it feels like my stomach has dropped or something like I don’t….

Youth spoke of the exhaustion, both physical and mental, which for many was tied to overthinking—a key characteristic of anxiety identified by youth in the study. A 13-year-old youth explained “when I come home from school my brain has already run a marathon and if I haven’t physically done something like that.” Similarly, an 11-year-old participant shared:

…like just imagine something that makes you really you know just low…. That’s what it feels like you’re slowly collapsing…. Kind of like if you’re made out of sugar and it’s raining or after you get a sugar high and then you start crashing, that’s what happens to me, I get really angry and I put all my energy into fighting and then I sink down.

Despite experiencing these bodily symptoms, youth nonetheless tried to cope and manage their symptoms by “pushing through”, challenging themselves to live and to participate in their daily lives as much as they could. A 16-year-old shared: “Like if you’re already being super anxious all the time keep doing it [pushing through] because it means you’re doing it right.” Similarly, another 16-year-old shared:

With anxiety, even if it’s light out, it is just complete darkness… It’s like water that keeps rising, it’s at my chin…Like I’m never out but just keep it from like below my chin instead of going into my mouth, so I keep struggling to paddle my way through.

### 3.3. The Metaphor of Lived Time: Play, Pause, Rewind, Forward 

**Figure 5:**
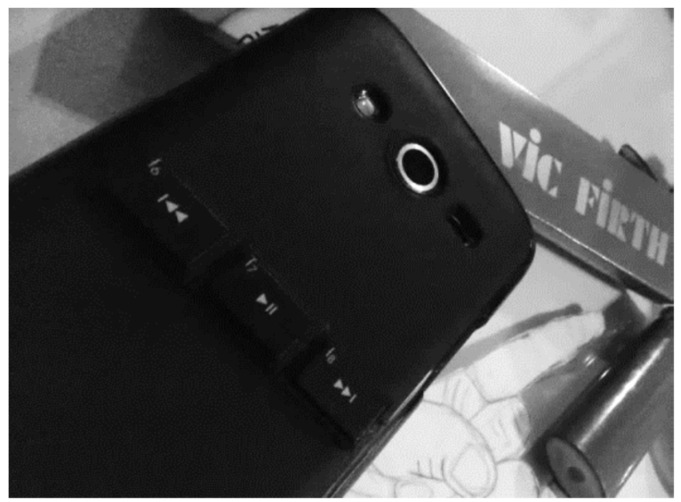
Play, pause, rewind, and forward.

Lived time can be understood as the subjective rather than “factual” experience of time; that is the way in which individuals temporally experience their lifeworld [88]. Overall, youth participants with anxiety described their challenges living in the moment, and how they often were stuck living in the past, or worrying about their future, and as such lived with little enjoyment of the present. A 16-year-old participant included a photo (Figure 5) submitted as part of the photovoice process that referenced her challenges living in the present:

I glued on keys from an old computer…. And I really liked the play pause, rewind and forward…. Just ‘cause it’s like, like start your life as in like play, pause and breathe…. Forward and think in the future…. And rewind and think in the past because that’s everything I kind of do…. Sometimes I just stop and breathe, sometimes I just you know go with it, sometimes I start thinking of good things that could happen in the future or bad things that could happen in the future and sometimes I start thinking of the past and sometimes it’s good or bad

**Figure 6:**
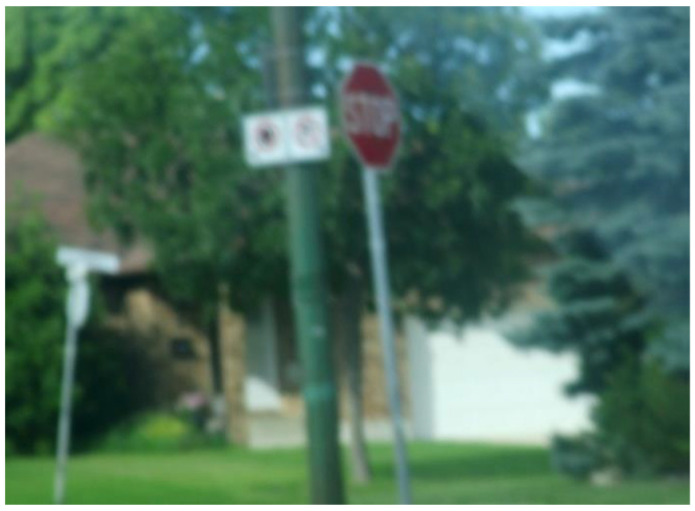
Image of stop sign submitted by youth participant.

In similar vein, a 16-year-old participant shared:

That’s the stop sign at the end of my street (Figure 6). And I just sort of thought like all of the times I feel like I’m just sort of standing and staying in one place, not really making progress in any field of my life… so that sort of represents anxiety and depression…. I’m not really happy being in that moment.

**Figure 7:**
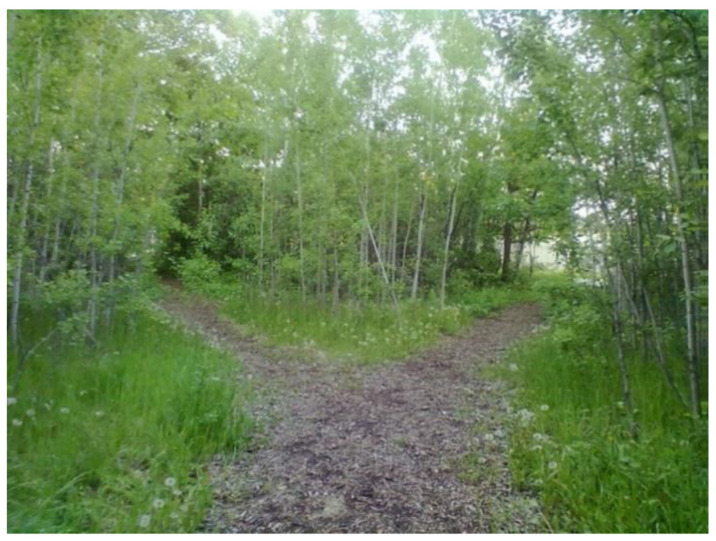
Image of pathways submitted by youth participant.

These feelings of being stuck in time, and in place, were often accentuated when youth had to make decisions. Living in the past, while fearing for the future, often made decision making in the present difficult for youth participants. A 22-year-old participant shared the following photograph (Figure 7) that she submitted for the project and shared a narrative that highlighted her difficulties in making decisions and the ways in which anxiety interacted with the decision-making process:

If you notice, there’s two pathways… So even though I’m going out to nature I feel better, as soon as I approached this almost like a fork right…I start feeling anxious again…. Like what way do I take?... Which always represents me in life, there’s, whenever I’m facing a decision it’s one of the worst causes of my anxiety because the what if comes up…. What if I take the wrong path and get lost or, or end up you know causing more problems or you know this and that and you know so many things come up, even though it’s such a beautiful scenery and it’s supposed to make me feel good, it’s just, I stood there going, which way, I mean even though it’s no big deal, I’m just taking a walk, all of a sudden I just thought of wow, like anything that gives, makes me decide…It just it feels horrible inside…I hate deciding.

For many youth participants, anxiety resulted in a cyclical pattern that took up a significant amount of time. For instance, a participant described living with anxiety as being akin to listening to a “bad record on repeat”. Time factored into the ways they described and conceptualized both their symptoms and experiences of living with anxiety. For instance, many participants submitted photographs of clocks on the wall and alarm clocks as a literal description time as a source of anxiety. However, for many other youth, submissions of clocks were more metaphorical, to represent how anxiety informed and controlled their experience with lived time, as well as how their lived time informed their anxiety. Other youth submitted examples of pacing as a literal and metaphorical description of their experience of lived time. This emphasis on time was something we incorporated into some of our knowledge translation products, in one instance having a ticking clock as the background noise to our film *Can’t You See I’m Struggling* [87].

While struggling with lived time, youth actively worked to be present in the moment. For instance, youth shared anecdotes of struggling with seasonal affective disorder in the winter months yet attempting to enjoy the sight and beauty of the snow falling on tree branches. Youth also attempted to manage living in the moment by envisioning a different and more positive path. A 16-year-old participant highlighted the importance of using a metaphor of plants regenerating in spring to describe in detail her own possibilities for personal growth and hope for the future, making it easier for her to live in the present:

When the snow melts like automatically expect to see like flowers and green grass and leaves on trees and then I always forget when the snow melts it’s like a long process of everything growing again and it was also kind of like this deeper message to me that you know when the snow melts you can’t automatically expect things to get better right away but like it’s a, like it’s a slow process and as long as you focus on the fact that like things will grow again..…So I feel if you connect yourself to a flower, like you’re dead under this miserable ice and it is like when the ice melts you’re still dead but you can teach yourself to grow again and it’s like I think that’s a really important metaphor.

### 3.4. The Metaphor of Lived Relations: A Fine Balance

**Figure 8:**
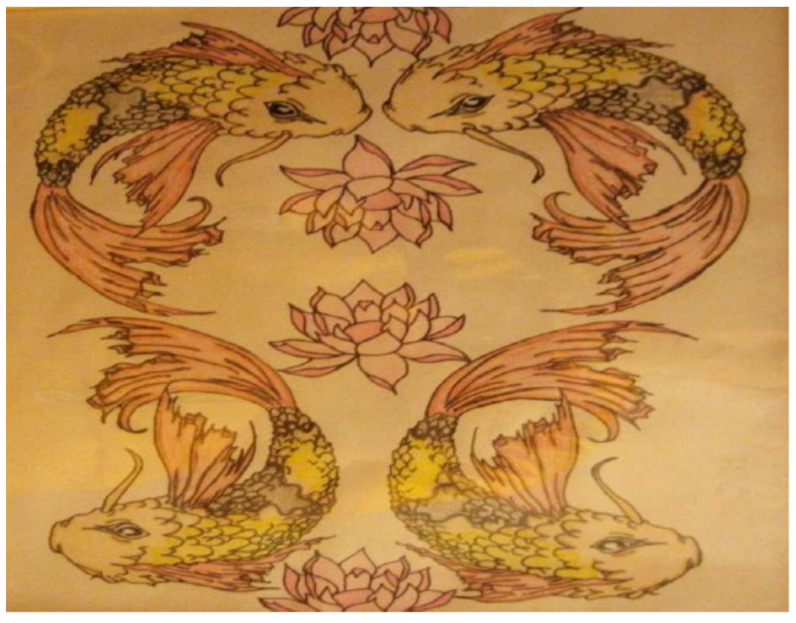
“Two sides of me”.

Lived relations refers to the subjective experience of relationships developed and maintained within our lifeworld [88]. Anxiety greatly impacts the lived relationships of young people. Overall, youth described a dilemma of being at once close to, and yet away from, others. A 22-year-old participant submitted a photograph (Figure 8) taken of her artwork to demonstrate the challenges in her lived relations resulting from her anxiety:

At times my anxiety can be represented in a way by those *sakura* which is cherry blossoms…. And it can be to the point where I can’t talk to people…So there are times where I can approach someone and be with them and, and go about my daily things, but after that the cherry blossoms seem to push me away which is like the anxiety makes me feel like I want to get away from people and it’s too much to handle and so it’s just like its two sides of me (chuckle).

**Figure 9:**
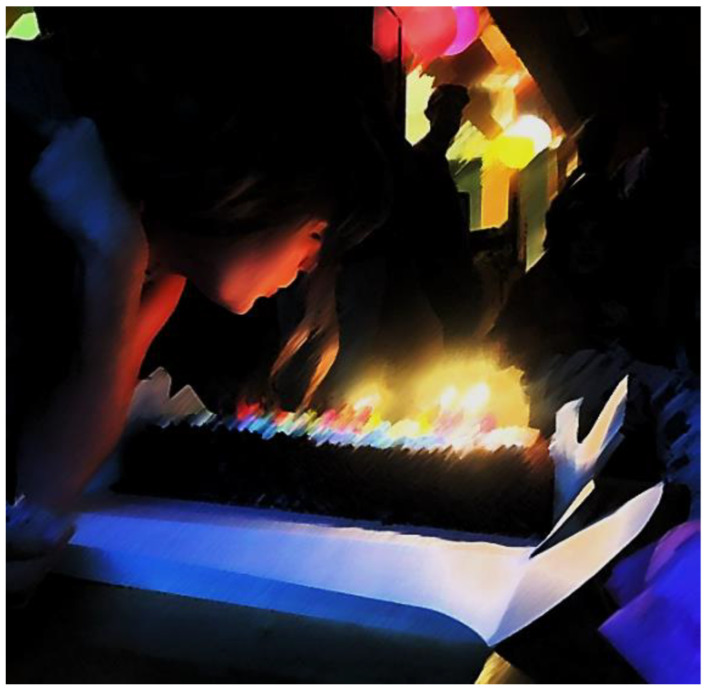
Image submitted by youth participant to demonstrate their anxiety over “being seen”.

Relationships for youth highlighted the difficulty that many youth in the project experienced in manoeuvring the relationships they had with others in their lives. One youth provided an example of a “good day” sharing a photograph (Figure 9) from her birthday party, surrounded by the people she loves. However, this good day was also a significant source of anxiety. The youth shared that having people watch her as she blew out her birthday candles also symbolized her anxiety—of “being seen” by others and having the attention focused on herself.

For other young people, there were times when they needed to be by themselves in part as an attempt to cope with their anxiety. They described feeling overwhelmed by the anxiety that came from navigating their social worlds, and how they would instead choose to close off to others. Photos were also submitted by young people to represent this shutting down to others. Many participants also submitted images of their phones as both a literal and metaphorical description of their anxiety and how it shaped their lived relations with others. For many youth, the telephone represented a source of their anxiety, and they described their fears of talking on the phone, but also the pressure they feel to constantly be connected particularly in the digital age. However, other youth also shared images of telephones more as a metaphor—to describe their fear of the unknown in the context of relationships—of not knowing how an interaction with others would play out and their anxiety of being around others. 

Some young people shared that at times, they felt very alone in their experiences of anxiety, and that they had no one there for them. A 16-year-old participant described:

[People] ask you what is like, cause I always get asked, what is anxiety like, I just, I just reply its hell…. It’s like you’re trapped in a world and no one’s there for you, yet they may be but you can’t see them, they can see you but you can’t, it’s like you can’t hear them but like they can’t hear you…. It’s like you want, it’s like they can, like maybe, maybe they can hear you but you just can’t hear them responding saying I’m here to help, I’m here to help.

For many youth, these feelings of being alone in their experiences were a key motivation for participating in the project, and this influenced our decision to create knowledge translation products such as these films, to show that youth were not alone.

**Figure 10:**
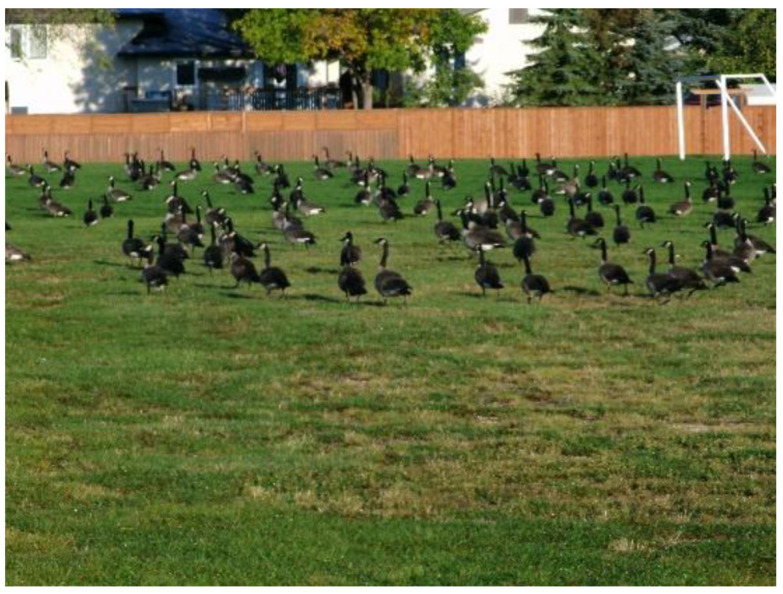
Photograph submitted by participant to represent anxiety over crowds.

Youth also utilized metaphors as a way to describe the manifestations and sources of their anxiety, such as the following from a 17-year-old participant:

Oh, this photograph (Figure 10) is technically a picture of a whole bunch of geese…But what I did with this one is I used the geese as a representation. First of all there’s geese everywhere… there was just too many of them so this one I used to represent crowds…. Because I didn’t want to go risk it in the shopping mall or like after a concert or a club or anything, so I thought geese were a safe way to go…. What I really liked about it is that when you’re anxiety and you’re in a crowd you don’t really notice each person individually or you can’t really tell the difference between each person, only know if it’s a person you don’t know and that causes anxiety. So, using geese where they almost all look the same is kind of the best analogy of what it’s like in the eyes of someone with anxiety in a crowd.

**Figure 11:**
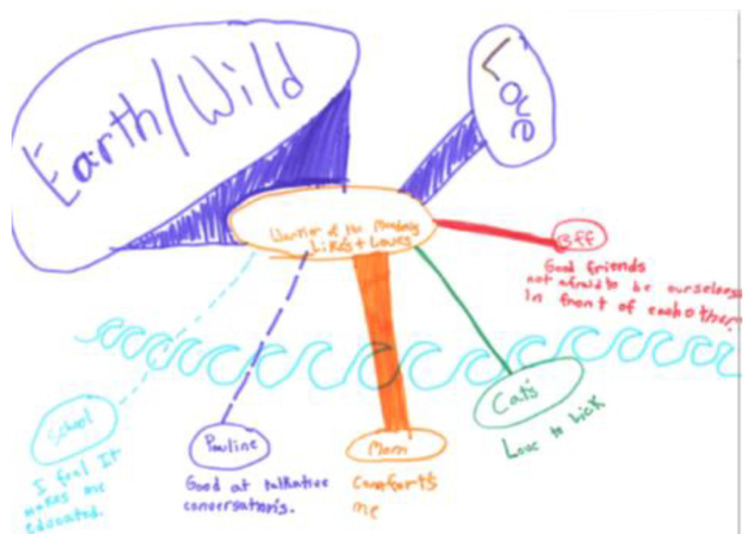
Ecomap from youth participant.

Young people also spoke about the relationships they had with themselves, which for many of the youth participants also entailed a relationship with their anxiety. Some participants also reflected on anxiety as their “friend” as contributing to who they are. Comfort and coping for many youth, comes from their relationships. As represented in many of the youth’s ecomaps, however, relationships were not limited to human relations but rather included objects (e.g., teddy bears, pets, nature). A 13-year-old participant highlighted some of these non-human elements in her ecomap (Figure 11), with a large node with solid lines denoting a positive relationship for nature, emotions, and her pet. Broken lines for this participant represented her notions of “pushing through”—of those aspects of life that were a source of anxiety but that also had positive aspects (e.g., participating in the research project).

**Figure 12:**
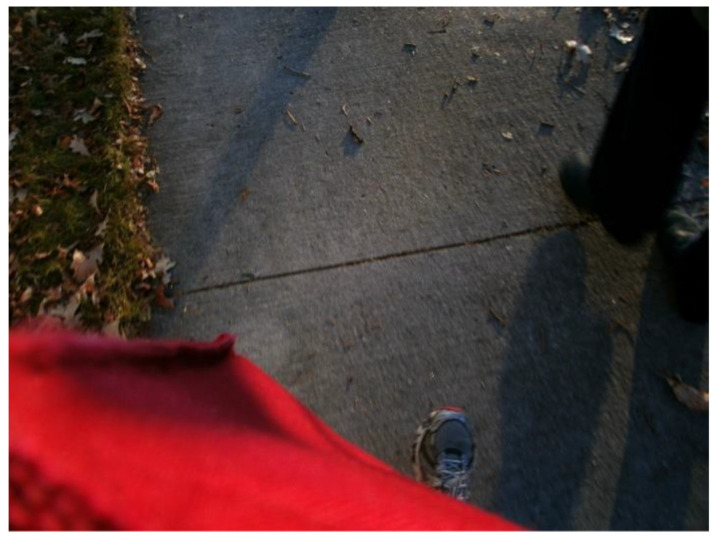
Image submitted by youth participant representing “small steps”.

Lived relationships also were a crucial part of the coping strategies identified by youth. When we asked youth to share their ideas of a “good day”, many youth talked about the ordinary and mundane—of spending time with those close to them and of having days when their anxiety was less impactful and more manageable. A 13-year-old participant shared the following image (Figure 12) and explained:

**Participant**: This is us walking to school… My mother and I….

**Interviewer:** And when you look at this picture what does it make you think or what does it remind you of?

**Participant:** Small steps…Just take it slow, small steps again.

### 3.5. Lived Meaning

Considerations of lived meaning emphasize subjectivity—to document how individuals experience and understand their world as real and meaningful [64]. Overall, young people described their anxiety using the metaphor of a monster—permeating all aspects of their lives, and contributing to feelings of fear, of loss, and of pain. An 11-year-old described:

But anxiety is like a monster it just creeps up on you…I see kind of see really kind of evil and red swirls…It kind of reminds me of the devil honestly, just a bunch of red swirls. Sometimes I tell the monster, like I don’t care, go bug someone else, not me right now…. o um I don’t know how old I was when I started, I think I was 10…. And I created mine, her name was Candy and she looks so sweet and beautiful and then she’s just like an evil monster.…. She [monster] came into my head all the time when I had anxiety and I would just say; go away, I don’t want to listen to you, so you need to leave…And then I would also like reach to my head and go like that, like I was pulled her out or something… And then I would just, I would lock my brain [motions as if turning a key] and be like okay you can’t get into my head now…I don’t know what, it just helped with like, oh she’s not in my head anymore, oh whoa and then I’ll just get distracted by something else.

**Figure 13:**
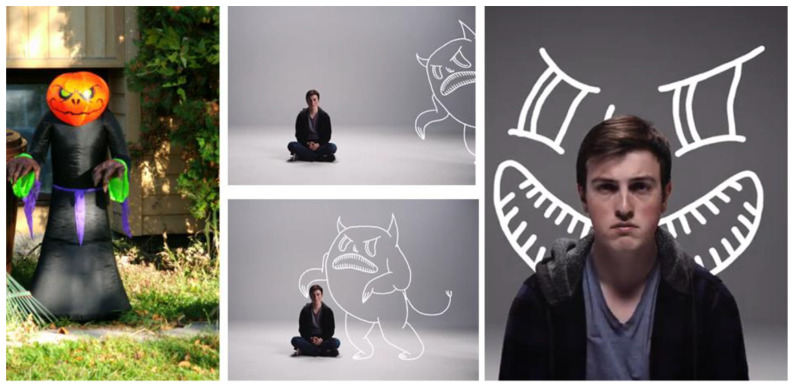
Anxiety as the Monster, as exemplified by a youth participant’s photovoice submission and as interpreted in our film series.

Similarly, an 18-year-old submitted a photograph (Figure 13) of a monster with the following description that was later incorporated into our film series [86]:

This one is a picture of what anxiety kind of sort of looks like in my head…It looks like a monster (chuckle)…. Especially when you don’t have any skills to deal with anxiety yet, it’s just a big bad scary monster…. It depends on the day you ask me, sometimes anxiety feels like a monster inside of me and sometimes anxiety, if I go without anxiety for a long time it will be like the monster is coming back to me.

**Figure 14:**
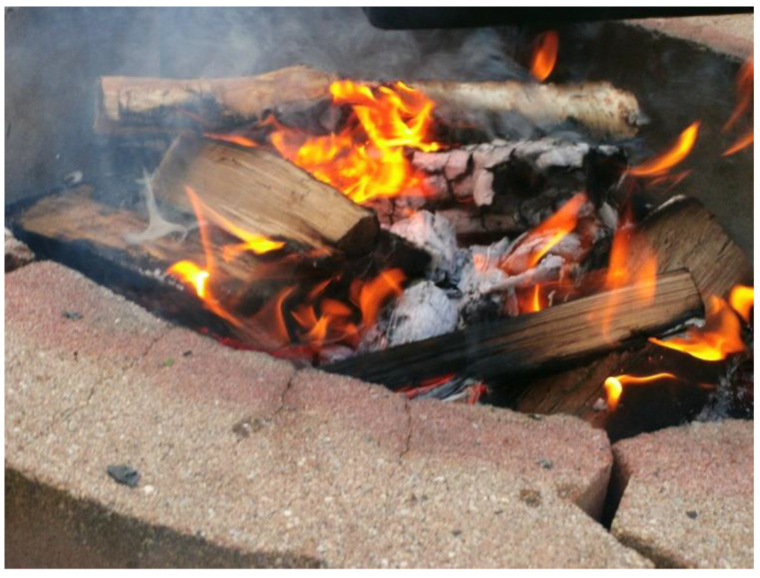
Image of fire submitted by a youth participant.

The overwhelming nature of this anxiety was not always recognized by others who interact with youth with anxiety, including by those working in the healthcare and education system, as well as by family members and close friends. Despite their challenging lived experiences, there were many glimmers of hope for youth and demonstrations of their strength. Youth spoke about learning and educating themselves on their anxiety as well as ways to manage it so that they could more actively participate in their lives. An 18-year-old participant shared the following photograph (Figure 14) and description:

This one [photograph] is a fire. I was having it at my lake but I thought it was a good picture because I think it represents anxiety pretty well… Anxiety could be provoked by the smallest little thing and it could turn into something big and ugly, so kind of like the smallest little spark can start a fire, but I also put it in the fire pit because what people think that I need to learn is how to control it or how to harness it to do something good. Like I know when I sometimes get anxious I can use that energy positively when I go work out so then I can work out longer or sometimes I use that to focus on something that I really need to do and I thought that was a good way of describing what can happen with anxiety. Another thing that can happen with anxiety if we don’t control it can be damaging to us and anything around us, just like fire can be damaging.

## 4. Discussion

There is an abundance of research aiming to understand anxiety and its comorbidities as clinical diagnoses, but the existential and social resonance remain underappreciated in the literature. There are only a few studies that have utilized a similar phenomenological approach to capture existential aspects of the illness experience for youth with anxiety, namely Woodgate et al.’s (2020) study of the sense of self in anxious youth [89] and Leone et al.’s (2013) study of anxious youth in high school [90]. Put together, the previous studies attest to the pervasiveness of anxiety across several, if not all, dimensions of youth’s lives, consistent with the findings of this study. The majority of the literature continues to be specific, for instance, social anxiety and its environmental (e.g., schools, clinics) and relational (e.g., peers, parents) aspects [91,92]. The adoption of an existential lens, used in conjunction with evidence-based measures, can provide access to the lived space, time, body, and relationships of youth with anxiety across populations and contexts. Visual art methods, such as photovoice, can aid the adoption of an existential lens as they are designed to align with a context and support a “re-visioning” of an experience [93,94], moving beyond the act of simply sharing to a more comprehensive shaping of youth’s mental illness experiences [95]. The photographs that emerged from this study were a form of metaphor making, meaningful on their own while also complementing the patient’s illness narratives.

Narrative metaphors contributed to the representation of the spatial experience; some youth described feeling “lost” in an expansive space, whereas others described a feeling of a shrinking space, with many feeling a lack of stability in their environment. This study also helped to provide insight into the multitude of environmental influences on the experience of anxiety, ranging from varied sensory inputs to personal situational meanings. Photovoice was especially helpful in conveying the often-intangible emotional aspects tied to their experiences of their lived space, echoing both feelings of powerlessness and resilience. This study’s use of photovoice has methodological implications as it exemplifies how visual art can be used as a metaphor to contribute to spatial gestalts that are a more accurate and complete representation of the participant’s lived space. Spatial metaphors are a key window for researchers, the public, and healthcare providers to understand the lived world of youth with anxiety, which can highlight ways to make spaces “safer” and inversely identify triggers and targets for therapy.

Youth’s descriptions of the embodied experience of anxiety were visceral and raw, and they relied on physical analogies and narrative metaphors to create understanding of their internal experience. Symptoms of anxiety were pervasive in their physical and psychological health, as well as their overall sense of well-being, attesting to the multidimensionality of the experience. The diversity and range of youth’s embodied experiences captured by narrative metaphors is irreducible; despite having a common diagnosis, this has important implications for healthcare providers. Although quantifiable and observable measures, such as questionnaires and scales, need to inform the assessment of anxiety in youth, they are insufficient when used alone and do not always capture the high level of comorbidity (e.g., between anxiety disorders, with depression) [96]. When used properly, metaphors can be a valuable tool for youth to convey what their experience is really like and can better inform an assessment of the impact of anxiety in their everyday life.

Metaphors helped to provide insight into the varied perception of time for youth with anxiety, ranging from an accelerated or a slowed sense of time, and even a sense of stopped time. In addition, the temporal distance between the “felt” time and the real time may also vary, with youth finding themselves thinking about the past or foreseeing the future. Both are equally anxiety-evoking, the future in its unpredictability and the past due to previous negative experience and the fear of re-living them. Living in the moment is a challenge for youth with anxiety, as many find themselves ruminating over previous or anticipated events in times.

Youth with anxiety often described social relationships and interactions as a “push–pull” phenomenon; occasionally anxiety-producing, yet also providing an important source of comfort and coping. This two-fold perspective on relationships resulted in youth carefully manoeuvring their social world, trying to balance risk with benefits. Additionally, many youth found themselves feeling alone, sometimes even in the presence of others. Metaphors can be a valuable way for youth to share their experiences with others, fostering connection and understanding, especially if the experience of anxiety is outside of that individual’s frame of reference. It is important to recognize that the social world of youth with anxiety is evolving and influenced by their many factors, including their internal state and external factors. Only recently have youth voiced their own experiences of the social and emotional aspects related to their social fears and anxieties [91]. For this reason, asking a young person how they want to be supported in that moment in time (e.g., silence in companionship, distraction, alone time) and recognizing that different forms of relations may be preferred (e.g., online groups, pet therapy) may be most beneficial. Time spent in nature has also been identified as a key element for young people in their coping and management [97].

### 4.1. Limitations

Though the goal of purposive sampling is to maximize variety or diversity [69,98], participants in this study were predominantly white and female, limiting transferability of findings to other populations. Future work should include more diverse groups of youth in order to broaden our understanding of the communication patterns and lived experiences of youth with anxiety disorders. Studies examining metaphor use in diverse groups have the potential to explore how different communication barriers (e.g., language, culture, gender, health literacy) can hinder or promote the utility of metaphors as a communication tool across different youths with anxiety.

### 4.2. Implications for Practice

The findings from this study provide support for the use of metaphors as a communication tool useful in facilitating healthcare communication, especially for youth with anxiety, and reducing knowledge translation barriers. Communication in healthcare is a characterized by cross-cultural interactions (e.g., age, social class, professional background) [31] and an epistemic imbalance between the patient and the healthcare provider [99]. Culture denotes the cognitive schemata a group of people use to make sense of new information [100]. Diagnostic tools and the use of language within such tools may be inadequate in actively drawing out from young people the full extent of their lived experience. When used in healthcare discussions, explanatory metaphors aim to cue shared cultural models in order to serve as a “bridge between expert and public understandings” [25]. Every mental illness experience, especially anxiety [13,89] and depression [12,101,102], is unique to the individual and embedded in their context. For this reason, a clinician’s use of metaphor should be tailored to the patient’s context and individual characteristics (e.g., cultural values, background, illness type and stage) [17,31,36]. By attending and responding to a patient’s chosen metaphors, a clinician stands to gain a deeper understanding of the many languages of mental illness and, in particular, their patient’s language. In addition to being mindful of the language in patient interactions, there is also a need to further educate the public around conceptualizations of mental health, particularly youth’s mental health literacy regarding symptoms of depression and anxiety [103,104,105]. In order to effect change, knowledge from health research must be articulated in a manner that makes it available and applicable [106,107]. Photovoice as a form of visual metaphor is a valuable method of making inherently personal experiences of illness accessible to others and enabling to rewrite social attitudes and beliefs about mental health [56,108,109].

## 5. Conclusions

To our knowledge, our study is the first to reveal that youth use metaphors to make sense of living with anxiety. Metaphors helped to provide insights into youth’s varied perspectives of their lived space, lived body, lived time, and lived relationships. Through the use of qualitative, arts-based research methods, youth were afforded the opportunity to express their existential challenges metaphorically. As Galasiński notes, a greater presence of qualitative research is needed in the areas of mental health research and psychiatry, considering qualitative research can result in the understanding of the meanings exchanged in the diverse contexts of social life, as well as offer insight into the complexity of clinical communication, the here-and-now of the clinical encounter [110]. We suggest that departing from the literal use of words through metaphors and utilizing qualitative, art-based methods can offer new opportunities for improving the therapeutic conversations and interactions clinicians have with youth experiencing mental illness [111].

## Supplementary Materials

The following are available online at https://www.mdpi.com/article/10.3390/ijerph18084315/s1.
