# Supplementary material for: Understanding Youth’s Lived Experience of Anxiety through Metaphors: A Qualitative, Arts-Based Study
Source: Int J Environ Res Public Health. 2021 Apr 19;18(8):4315. doi: 10.3390/ijerph18084315 (PMC8074263; doi:10.3390/ijerph18084315)
Supplement: Supplementary file 1 [file ijerph-18-04315-s001.zip › ijerph-1121245-supplementary.pdf]

Supplementary Materials

Video 1. Hiding the Struggle, Part 1 of A Day in the Life of a Young Person with Anxiety: www.youtube.com/watch?v=tunX4ohxoqc&t=32s

Video 2. Fear of the Unknown, Part 2 of A Day in the Life of a Young Person with Anxiety: www.youtube.com/watch?v=I1IKeNbHdOs&t=57s

Video 3. Taking Up Space, Part 3 of A Day in the Life of a Young Person with Anxiety: www.youtube.com/watch?v=ic4thVMjUVM&t=12s

Video 4. Feeling Different, Part 4 of A Day in the Life of a Young Person with Anxiety: www.youtube.com/watch?v=XOqSqPkvEjM&t=5s

Video 5. Can't You See I'm Struggling, Part 5 of A Day in the Life of a Young Person with Anxiety:
www.youtube.com/watch?v=EqD_-JzVABw

Video 6. The Monster, Part 1 of Youth Voices: What it is like to live with anxiety: www.youtube.com/watch?v=F9MjseqVhFc

Video 7. Twenty Different Emotions, Part 2 of Youth Voices: What it is like to live with anxiety: www.youtube.com/watch?v=fYIZCKKB-bc

Video 8. Overthinking, Part 3 of Youth Voices: What it is like to live with anxiety: www.youtube.com/watch?v=3fpsFfl7GBI&t=78s

Video 9. Trapped in an Empty World, Part 4 of Youth Voices: What it is like to live with anxiety: www.youtube.com/watch?v=EzpZ6EEtjhc&t=33s

Video 10. Fighting to Stay on the Path, Part 5 of Youth Voices: What it is like to live with anxiety:
www.youtube.com/watch?v=oHOfCYcwKJI&t=61s

Zine 1. Youth Voices: Their Lives and Experiences of Living with an Anxiety Disorder. An online photo exhibit. https://online.flowpaper.com/7c590787/YouthVoices/#page=1
